# Fatty Acid Signatures as Nutritional Indices: Molecular Gateways to Health, Aging, and Functional Food Innovation

**DOI:** 10.1002/fsn3.72284

**Published:** 2026-08-26

**Authors:** Mohammad Nazrul Islam Bhuiyan, Md. Selim Reza, Farzana Mim

**Affiliations:** ^1^ BCSIR Chattogram Laboratories, BCSIR Chattogram Bangladesh; ^2^ Institute of Food Science and Technology, Bangladesh Council of Scientific and Industrial Research (BCSIR) Dhaka Bangladesh; ^3^ Department of Biochemistry and Molecular Biology Jahangirnagar University Savar Bangladesh

**Keywords:** artificial intelligence, fatty acid signatures, foodomics, functional foods, functional lipidome, healthy aging, lipidomics, multi‐omics, precision nutrition

## Abstract

Fatty acid signatures (FA signatures), defined as the relative distribution of saturated, monounsaturated, and polyunsaturated fatty acids within specific biological compartments, have emerged as integrated indicators of dietary lipid exposure, endogenous metabolism, and tissue‐specific lipid remodeling. Rather than serving solely as biomarkers of nutritional intake, FA signatures influence membrane architecture, cellular signaling, inflammatory regulation, mitochondrial function, and gene expression, linking dietary lipid quality with metabolic health, chronic disease susceptibility, and biological aging. This review critically synthesized current evidence on the biosynthesis, regulation, analytical assessment, and biological significance of FA signatures, with particular emphasis on cardiometabolic health, neuroinflammation, cognitive aging, and healthy longevity. We distinguish the complementary roles of conventional fatty acid profiling and high‐resolution lipidomics in resolving fatty acid composition and molecular lipid species, while highlighting persistent analytical challenges, including biological matrix specificity and the limited resolution of positional and geometric fatty acid isomers. We further examine how integration of lipidomics with nutrigenomics, metabolomics, foodomics, and emerging computational approaches can improve mechanistic interpretation, biomarker discovery, and precision nutrition. Collectively, FA signatures represent dynamic metabolic phenotypes that connect dietary exposure with membrane biology, metabolic regulation, and healthy aging, while providing a promising foundation for evidence‐based nutritional strategies and next‐generation functional food research.

## Graphical abstract

Systems framework linking dietary fatty acids with the functional lipidome, health, and precision nutrition. Dietary fatty acids from plant‐based, marine, and animal sources undergo absorption, endogenous desaturation and elongation, oxidation, and tissue‐specific incorporation and remodeling, generating individualized FA signatures. These signatures integrate classical FA‐derived indices with molecular lipid species and functional outcomes, while being shaped by genetic, epigenetic, microbiome, lifestyle, and environmental factors. Integration with genomics, transcriptomics, proteomics, metabolomics, lipidomics, and foodomics, supported by computational and AI approaches, enables mechanistic interpretation of lipid remodeling and its associations with cardiometabolic health, inflammation, brain function, healthy aging, and longevity. This systems‐level framework provides a basis for biomarker discovery, precision nutrition, functional‐food evaluation, and preventive health strategies.

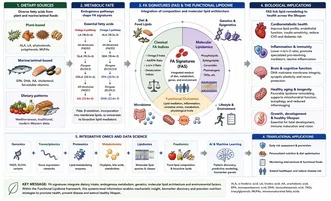

## 1 Introduction

Fatty acids are fundamental constituents of biological membranes, where they are incorporated predominantly into phospholipids and other complex lipids that determine membrane architecture, biophysical properties, and cellular function. The relative abundance and molecular distribution of saturated fatty acids (SFAs), monounsaturated fatty acids (MUFAs), and polyunsaturated fatty acids (PUFAs) influence membrane fluidity, curvature, permeability, lipid‐raft organization, membrane‐protein activity, and intracellular signaling. Membrane lipid composition is therefore a dynamically regulated component of cellular homeostasis rather than a passive reflection of dietary fat intake or an energy‐storage reservoir (van Meer et al. 2008; Ferreri et al. 2016; Levental et al. 2020).

This membrane‐centered perspective provides the biological basis for fatty acid signatures (FA signature), defined here as characteristic patterns in the relative distribution of fatty acids within specific biological compartments, including plasma lipids, erythrocyte membranes, adipose tissue, and tissue phospholipids. FA signatures integrate dietary exposure with endogenous lipogenesis, desaturation, elongation, β‐oxidation, lipid turnover, and membrane remodeling and can therefore provide information that extends beyond dietary intake assessment alone (Chen and Liu 2020; Almeida et al. 2021; Ademowo et al. 2024). Their interpretation, however, depends strongly on the biological matrix because circulating plasma lipids, erythrocyte membranes, adipose tissue, and tissue‐specific lipid pools differ in turnover rate, metabolic function, and physiological relevance (Ferreri et al. 2016).

Membrane fatty acids perform both structural and regulatory functions. Long‐chain PUFAs influence membrane organization, receptor activity, mitochondrial physiology, and the generation of bioactive lipid mediators, whereas excessive accumulation of certain saturated lipid species can contribute to lipotoxic stress and metabolic dysfunction under conditions of chronic energy excess. EPA, DHA, and arachidonic acid (AA) also serve as precursors for diverse families of lipid mediators involved in vascular regulation, immunity, inflammation, and inflammation resolution (Harayama and Shimizu 2020; Oppedisano et al. 2020; Schulze et al. 2020; Djuricic and Calder 2025). Thus, the physiological significance of an FA signature arises from the interaction between membrane structure, lipid metabolism, signaling pathways, and the metabolic context in which individual fatty acids are incorporated.

The biological relevance of FA signatures extends further to transcriptional regulation, mitochondrial metabolism, immune function, and epigenetic control. Fatty acids and lipid‐derived metabolites interact with nutrient‐sensitive transcription factors and signaling pathways that regulate substrate utilization, mitochondrial adaptation, inflammatory responses, and cellular stress. They may also influence DNA methylation and other chromatin‐associated processes, linking nutritional lipid exposure with longer‐term regulatory responses (Haro et al. 2019; González‐Becerra et al. 2019; Ediriweera and Gayashani Sandamalika 2025). Human studies similarly demonstrate age‐associated variation in circulating fatty acids and relationships between PUFA status, inflammatory balance, oxidative stress, and healthy‐aging phenotypes (Ali et al. 2023; Aiello et al. 2024; Li et al. 2025).

The emergence of lipidomics has broadened the interpretation of FA signatures by revealing the molecular lipid species in which fatty acids are incorporated. Rather than considering an individual fatty acid independently of its biochemical context, molecular lipid profiling can characterize phospholipids, sphingolipids, ceramides, plasmalogens, glycerolipids, and other lipid classes that participate in membrane organization and metabolic signaling (Gross 2017; Han and Gross 2022). This increased molecular resolution has contributed to the identification of lipid patterns associated with cardiometabolic risk, nutritional responsiveness, and healthy aging and has strengthened the transition from conventional lipid measurements toward systems‐level lipid phenotyping (Lauber et al. 2022; Eichelmann et al. 2024; Ademowo et al. 2024).

Parallel developments in foodomics have extended this molecular perspective to food and nutrition science. Advanced analytical workflows can characterize complex food lipidomes, evaluate changes induced by processing and extraction, and identify molecular lipid features relevant to nutritional quality, food authenticity, sustainability, and functional‐food development (Herrero et al. 2012; Mahato et al. 2024; Martakos et al. 2024). Recent characterization of marine‐derived lipid resources further illustrates how species and extraction procedures influence molecular lipid composition and antioxidant properties, emphasizing that the nutritional value of a lipid source depends on more than total fatty‐acid abundance (Martakos et al. 2026).

Taken together, current evidence supports a transition from interpreting FA signatures as simple indicators of dietary fat exposure toward viewing them as integrated metabolic phenotypes linking membrane architecture, endogenous lipid metabolism, nutritional status, molecular signaling, and physiological adaptation. Connecting established FA biomarkers with molecular lipidomics, foodomics, nutrigenomics, metabolomics, and aging biology may therefore improve mechanistic understanding of diet–lipid interactions and support the development of evidence‐based precision nutrition. Importantly, such integration should build on validated analytical and biological evidence rather than introduce unvalidated composite classifications.

## 2 Review Methodology

The literature synthesis was developed using a structured search and critical‐appraisal strategy covering fatty acid signatures, membrane lipid biology, lipidomics, nutritional biomarkers, healthy aging, precision nutrition, and functional‐food research. Relevant literature was identified from major biomedical and multidisciplinary databases, including PubMed, Scopus, Web of Science, and ScienceDirect. Priority was given to recent peer‐reviewed original studies, systematic reviews, meta‐analyses, and authoritative reviews, while seminal publications were retained when required to establish methodological or mechanistic foundations.

Search concepts included combinations of terms related to fatty acid signature, fatty acid profile, membrane fatty acids, lipidomics, dietary fatty acids, omega‐3 fatty acids, omega‐6 fatty acids, membrane lipids, healthy aging, biological aging, precision nutrition, foodomics, functional foods, nutrigenomics, metabolomics, and epigenetics. Literature was prioritized when it provided mechanistic information, clinically relevant biomarker data, longitudinal or intervention evidence, or integration of lipid measurements with complementary molecular or nutritional approaches.

Evidence was evaluated according to study design, biological matrix, analytical approach, population characteristics, and translational relevance. Particular attention was given to the compartment in which fatty acids were measured because plasma, whole blood, erythrocytes, adipose tissue, and isolated membrane lipids represent metabolically distinct pools with different turnover characteristics and biological interpretations (Ferreri et al. 2016). This distinction was considered when comparing findings across observational studies, intervention trials, and lipidomic investigations.

Analytical methodology was also considered during evidence appraisal because differences in sample preparation, extraction, derivatization, chromatographic procedures, structural annotation, and quality control can influence measured lipid profiles. Rather than repeatedly contrasting individual analytical platforms throughout the manuscript, methodological differences were interpreted according to the specific biological question addressed and are discussed in detail in the dedicated analytical section. Particular caution was applied when comparing studies involving structurally related or isomeric fatty acids because positional and geometric differences can carry distinct biological information (Sansone et al. 2013; Scanferlato et al. 2019; Ferreri et al. 2023).

The synthesis emphasized critical interpretation rather than exhaustive description. Findings were examined for consistency across populations and experimental settings, biological plausibility, methodological limitations, and evidence for association versus causation. Integration across nutritional biochemistry, membrane biology, lipidomics, foodomics, aging research, and complementary omics was used to identify robust areas of agreement, unresolved mechanisms, and priorities for translational research.

## 3 Molecular Language of Fatty Acids: Biosynthesis, Remodeling, and Cellular Signaling

Fatty acids are central components of cellular lipid metabolism, where their biological roles extend far beyond energy storage to include membrane organization, signaling, and metabolic regulation. Their composition is shaped by dietary intake, endogenous synthesis, desaturation, elongation, β‐oxidation, genetic variation, and continuous membrane remodeling. Understanding these interacting processes provides the mechanistic basis for interpreting FA signatures as dynamic molecular phenotypes linked to metabolic health, disease susceptibility, and biological aging.

### 3.1 Origins of FA Signatures: Integration of Diet, Genetics, and Endogenous Lipid Metabolism

**FIGURE 1:**
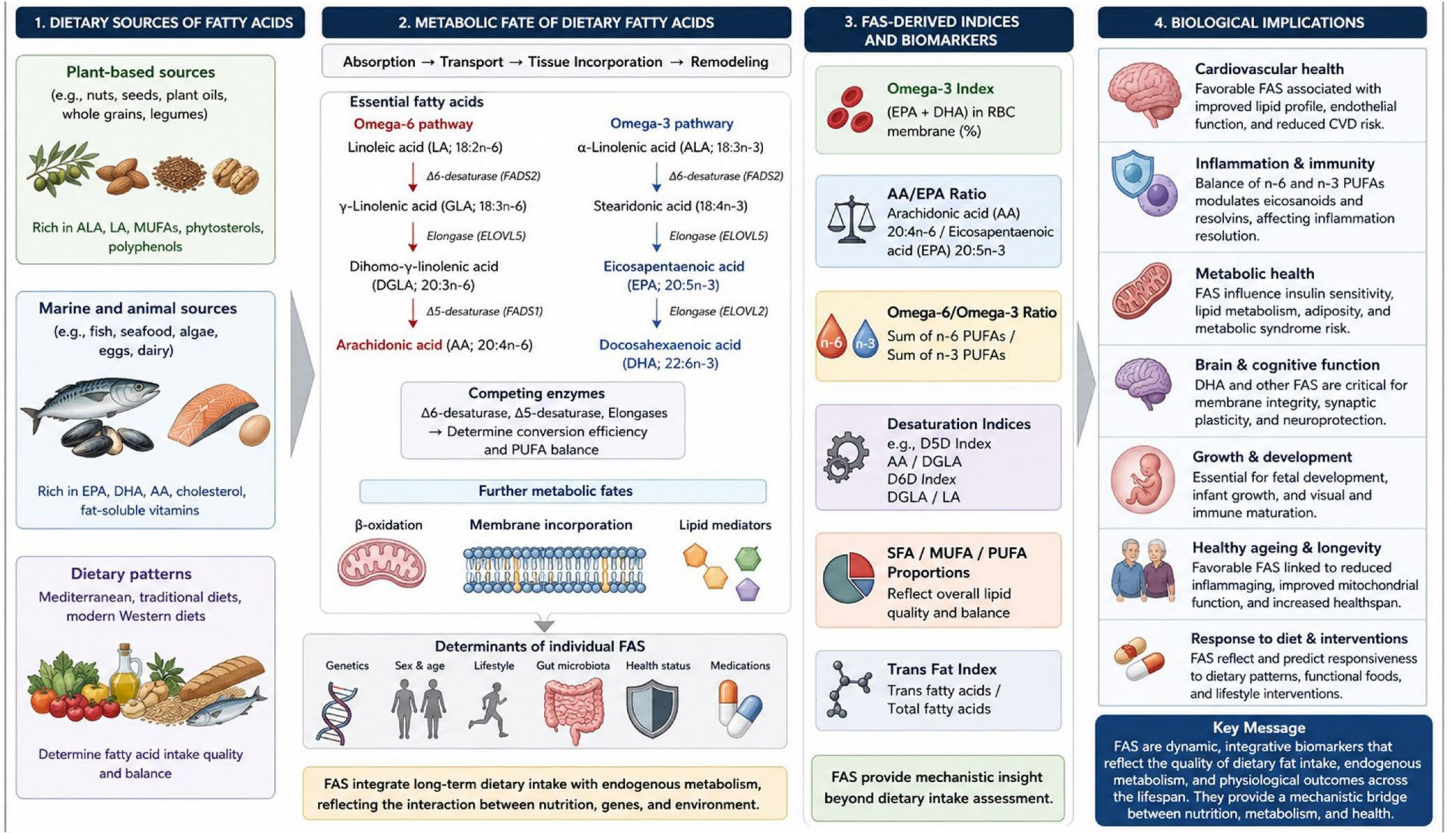
Conceptual overview of the biological origin and functional significance of FA signatures. Dietary saturated, monounsaturated, and polyunsaturated fatty acids interact with endogenous lipid metabolism through de novo lipogenesis, fatty‐acid elongation, desaturation involving FADS1/FADS2 and ELOVL enzymes, β‐oxidation, and membrane lipid remodeling to generate tissue‐specific FA signatures. Membrane phospholipids represent a particularly informative biological compartment because their fatty‐acyl composition integrates dietary exposure with endogenous metabolic regulation and tissue‐specific remodeling. Alterations in membrane lipid composition influence membrane fluidity, receptor organization, lipid‐raft dynamics, mitochondrial function, inflammatory signaling, and transcriptional regulation. Through these interconnected processes, FA signatures link nutritional lipid exposure with metabolic homeostasis, biological aging, and disease susceptibility and provide a mechanistic basis for their investigation in precision nutrition and functional‐food research.

FA signatures arise from the coordinated interaction of dietary lipid exposure, endogenous metabolism, genetic variation, and tissue‐specific lipid remodeling. They should therefore be interpreted as dynamic metabolic phenotypes rather than static reflections of dietary intake. Their composition depends strongly on the lipid compartment examined because fatty acids are distributed among phospholipids, triacylglycerols, cholesteryl esters, sphingolipids, and other lipid classes with distinct structural, storage, and signaling functions. Membrane phospholipids are particularly informative for investigating cellular lipid homeostasis because their fatty‐acyl composition is actively regulated and directly influences membrane biophysical properties and signaling processes (van Meer et al. 2008; Ferreri et al. 2016; Levental et al. 2020). The principal determinants contributing to the formation of tissue‐specific FA signatures are summarized in Table 1 and illustrated in Figure 1.

**TABLE 1:** Major enzymatic regulators governing FA signature composition and membrane lipid remodeling. Endogenous fatty acid metabolism is regulated by coordinated activities of desaturases, elongases, and de novo lipogenesis pathways that determine the relative abundance of saturated, monounsaturated, and polyunsaturated fatty acids within membrane phospholipids. These enzymatic processes integrate dietary lipid availability with genetic and metabolic regulation, thereby shaping FA signature and influencing membrane homeostasis, inflammatory signaling, and metabolic health.

Enzyme/Pathway	Primary biochemical function	Major regulators	Contribution to FA signatures and membrane lipid remodeling	References
Fatty acid desaturase 1 (FADS1)	Δ5‐desaturation of polyunsaturated fatty acid (PUFA) intermediates	Dietary PUFA availability; FADS genetic polymorphisms	Regulates long‐chain omega‐6 and omega‐3 PUFA biosynthesis, membrane phospholipid composition, and inflammatory balance	Chaaba et al. (2023); Wang et al. (2023); Mariamenatu and Abdu (2021)
Fatty acid desaturase 2 (FADS2)	Δ6‐desaturation of linoleic acid (LA) and α‐linolenic acid (ALA)	Dietary PUFA intake; FADS genetic polymorphisms	Controls conversion of essential fatty acids into long‐chain PUFAs and influences the omega‐6/omega‐3 balance	Chaaba et al. (2023); Mariamenatu and Abdu (2021); Wang et al. (2023)
Elongation of very long‐chain fatty acids protein 2 (ELOVL2)	Elongation of C20–C22 PUFAs during docosahexaenoic acid (DHA) biosynthesis	PUFA availability; peroxisome proliferator‐activated receptor (PPAR) signaling	Supports DHA biosynthesis, membrane phospholipid remodeling, and maintenance of membrane function	Wang et al. (2023); Harayama and Shimizu (2020)
Elongation of very long‐chain fatty acids protein 5 (ELOVL5)	Elongation of C18–C20 PUFAs	Liver X receptor (LXR) and PPAR signaling	Regulates long‐chain PUFA availability and membrane lipid remodeling	Wang et al. (2023); Harayama and Shimizu (2020)
Stearoyl‐CoA desaturase 1 (SCD1)	Conversion of saturated fatty acids into monounsaturated fatty acids	Sterol regulatory element‐binding protein‐1c (SREBP‐1c); insulin; carbohydrate availability	Regulates membrane fluidity, de novo lipogenesis, lipid storage, and metabolic flexibility	Haro et al. (2019); Schulze et al. (2020); Wang et al. (2023)
De novo lipogenesis (DNL)	Synthesis of palmitate from excess carbohydrate	Insulin; carbohydrate excess; SREBP‐1c	Promotes saturated fatty acid accumulation, membrane lipid remodeling, and metabolic dysfunction when chronically activated	Haro et al. (2019); Schulze et al. (2020)

Dietary fatty acids provide substrates for energy metabolism, lipid storage, and structural membrane remodeling. Following digestion, absorption, and systemic transport, SFAs, MUFAs, and essential PUFAs can be oxidized, stored, or incorporated into complex lipids. Linoleic acid (LA; 18:2 n‐6) and α‐linolenic acid (ALA; 18:3 n‐3) undergo sequential elongation and desaturation through partially shared enzymatic pathways, contributing to the synthesis of longer‐chain metabolites, including AA, EPA, and DHA (Harayama and Shimizu 2020; Rincón‐Cervera et al. 2022). Conversion efficiency varies considerably among individuals and is influenced by substrate availability, enzyme activity, nutritional status, genetic background, and competition among fatty‐acid precursors.

The physiological implications of n‐6 and n‐3 PUFA metabolism cannot be reduced to a single dietary ratio. Biological effects depend on absolute intake, the identity of individual fatty‐acid species, endogenous conversion efficiency, tissue incorporation, and downstream mediator synthesis. Although dietary imbalance may alter substrate availability within these pathways, current evidence supports interpretation of n‐6 and n‐3 fatty acids according to both amount and metabolic context rather than assuming that n‐6 PUFAs are intrinsically adverse (Schulze et al. 2020; Cao, Yang, Guo, et al. 2024; Cao, Yang, McClements, et al. 2024; Djuricic and Calder 2025).

Endogenous metabolism further modifies dietary signals. De novo lipogenesis generates fatty acids from excess nonlipid substrates, whereas desaturation, elongation, β‐oxidation, acyl‐chain remodeling, and lipid turnover continually reshape cellular lipid composition according to metabolic demand. FADS1 and FADS2 contribute to PUFA desaturation, while members of the ELOVL family regulate fatty‐acid elongation and influence the synthesis and distribution of long‐chain and very‐long‐chain fatty acids (Chaaba et al. 2023; Wang et al. 2023). These pathways are central to the metabolic origin of FA signatures and are integrated within the conceptual scheme shown in Figure 1. Nutrient‐sensitive transcriptional pathways involving sterol regulatory element‐binding proteins and peroxisome proliferator‐activated receptors further coordinate fatty‐acid synthesis, oxidation, storage, and metabolic adaptation (Haro et al. 2019).

Genetic variation adds substantial interindividual heterogeneity to these pathways. Polymorphisms affecting desaturases, elongases, and related lipid‐metabolic proteins can modify circulating and membrane fatty‐acid composition and influence responsiveness to dietary interventions (Chaaba et al. 2023). Individuals consuming similar amounts of dietary fatty acids may therefore develop different biochemical FA signatures because dietary substrates are processed through genetically and metabolically distinct pathways.

Membrane remodeling provides an additional regulatory layer. Cells can preserve membrane function despite changes in dietary lipid availability by adjusting fatty‐acyl composition, lipid‐class distribution, and cholesterol–phospholipid interactions. Experimental evidence demonstrates that mammalian membranes actively compensate for dietary lipid perturbations through coordinated lipidomic and biophysical remodeling, emphasizing homeostatic control rather than passive incorporation of dietary fatty acids in fixed proportions (Levental et al. 2020). Consequently, FA signatures represent the integrated outcome of dietary exposure, absorption, endogenous metabolism, genetic regulation, tissue turnover, and membrane homeostasis.

Molecular lipid profiling has added important structural context to these processes by identifying the lipid classes and molecular species that undergo remodeling. This information is biologically relevant because the same fatty‐acyl chain may have different functional consequences when incorporated into phospholipids, sphingolipids, ceramides, plasmalogens, or storage lipids (Gross 2017; Han and Gross 2022). Detailed methodological considerations are addressed separately in Section 5, thereby preserving the biological focus of the present section and avoiding unnecessary repetition of platform‐specific analytical concepts.

### 3.2 FA Signatures as Structural Regulators, Signaling Mediators, and Epigenetic Modulators

**FIGURE 2:**
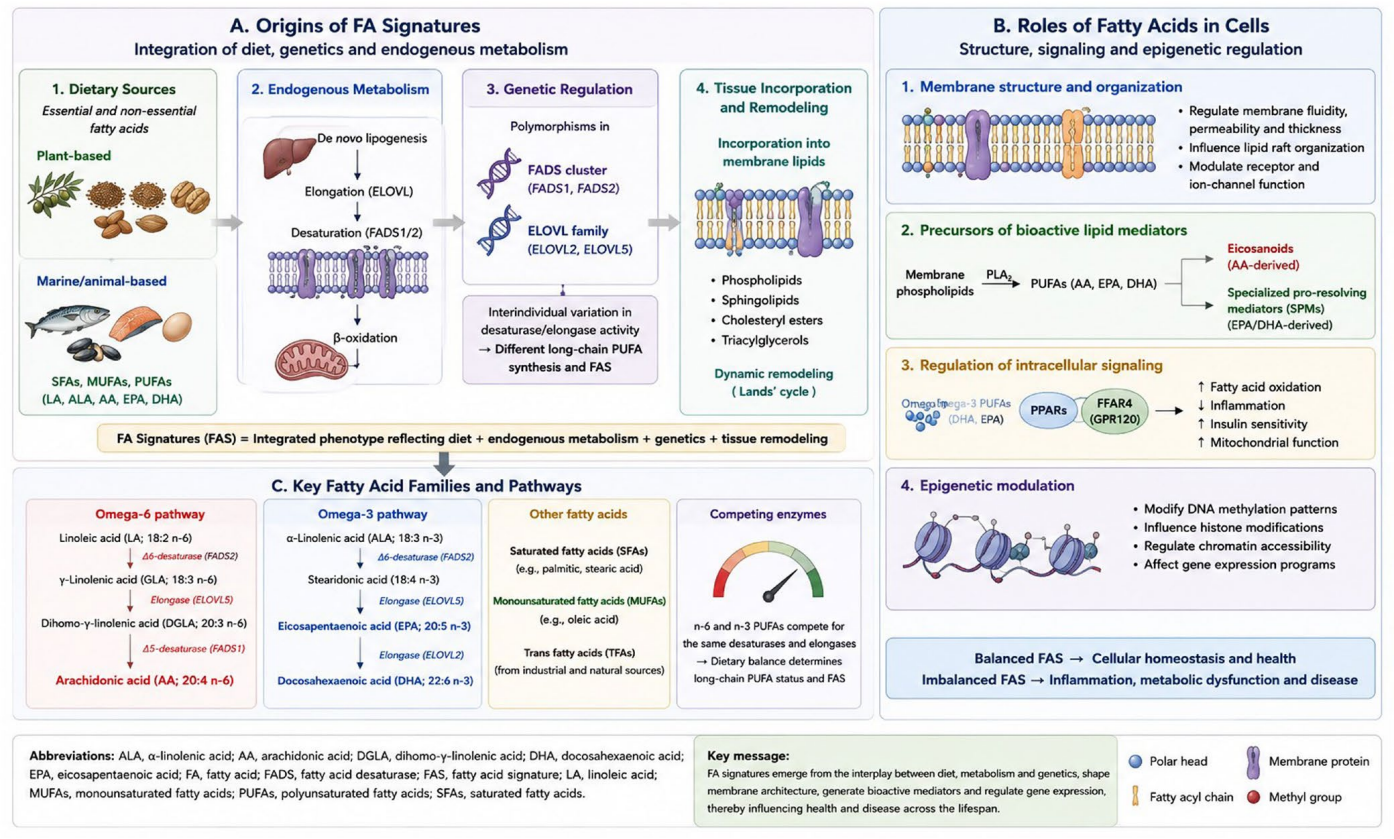
Mechanistic pathways through which FA signatures regulate cellular physiology and disease risk. Balanced membrane FA signatures maintain membrane architecture, intracellular signaling, and metabolic homeostasis. Following phospholipid remodeling, arachidonic acid (AA), eicosapentaenoic acid (EPA), and docosahexaenoic acid (DHA) generate distinct classes of bioactive lipid mediators that regulate inflammation and its resolution. Concurrently, fatty acids modulate nutrient‐sensitive signaling pathways, including PPARs, FFAR4 (GPR120), SREBP‐1c, and AMPK, influencing lipid metabolism, mitochondrial function, oxidative stress, insulin sensitivity, and epigenetic regulation. Coordinated alterations in these pathways contribute to cardiometabolic disease, neurodegeneration, and biological aging, illustrating how membrane lipid composition functions as a molecular interface between diet and cellular physiology.

Following incorporation into complex membrane lipids, fatty acids become important determinants of membrane organization and cellular regulation. Their effects depend on chain length, degree of unsaturation, double‐bond configuration, lipid‐class distribution, and interactions with cholesterol and membrane proteins. Regulation of these properties contributes to membrane fluidity, permeability, curvature, receptor mobility, ion‐channel activity, vesicular trafficking, and organization of signaling domains (van Meer et al. 2008; Ferreri et al. 2016; Levental et al. 2020). The principal signaling and regulatory pathways associated with FA signatures are summarized in Table 2 and illustrated in Figure 2.

**TABLE 2:** Major signaling pathways regulated by FA signatures and their biological significance. FA signatures influence multiple interconnected signaling pathways through their incorporation into membrane phospholipids and their conversion into bioactive lipid mediators. These pathways regulate membrane homeostasis, inflammatory resolution, energy metabolism, transcriptional regulation, and epigenetic programming, thereby linking dietary lipid quality with metabolic health, healthy aging, and disease susceptibility. High‐resolution lipidomics further demonstrates that these biological effects depend not only on fatty acid abundance but also on the molecular lipid species and lipid classes in which fatty acids are incorporated.

Signaling pathway	Principal lipid mediators/effectors	Primary biological function	Physiological and clinical relevance	References
Arachidonic acid‐derived eicosanoid pathway	Prostaglandins, thromboxanes, leukotrienes	Regulates inflammatory responses, vascular tone, platelet activation, and immune signaling	Cardiovascular disease, chronic inflammation, metabolic dysfunction	Harayama and Shimizu (2020); Oppedisano et al. (2020); Banaszak et al. (2024)
Specialized proresolving mediator (SPM) pathway	Resolvins, protectins, maresins (derived from EPA and DHA)	Promotes resolution of inflammation, tissue repair, macrophage efferocytosis, and restoration of homeostasis	Healthy aging, immune regulation, neuroprotection, inflammation resolution	Kumar et al. (2019); Oppedisano et al. (2020); Banaszak et al. (2024)
Peroxisome proliferator‐activated receptor (PPAR) signaling	PPARα, PPARγ and PPARδ activated by fatty acids and their metabolites	Regulates fatty acid oxidation, glucose metabolism, adipogenesis, and inflammatory gene expression	Metabolic homeostasis, insulin sensitivity, precision nutrition	Haro et al. (2019); Kumar et al. (2019); Yang et al. (2024)
Free fatty acid receptor 4 (FFAR4/GPR120) signaling	Eicosapentaenoic acid (EPA), docosahexaenoic acid (DHA)	Enhances insulin sensitivity and suppresses NF‐κB‐ and JNK‐mediated inflammatory signaling	Obesity, insulin resistance, type 2 diabetes mellitus	Yang et al. (2024); Oppedisano et al. (2020)
Epigenetic regulation	DNA methylation, histone acetylation, histone modification, acetyl‐CoA‐dependent chromatin remodeling	Regulates long‐term gene expression and metabolic programming in response to dietary lipids	Healthy aging, inflammation regulation, nutrigenomics, precision nutrition	González‐Becerra et al. (2019); Tremblay et al. (2017); Ediriweera and Gayashani Sandamalika (2025)
Membrane lipid remodeling and lipidomic networks	Phospholipids, sphingolipids, ceramides, plasmalogens, triacylglycerols	Maintains membrane homeostasis, lipid signaling, and metabolic adaptation through coordinated lipid remodeling	Biological aging, cardiometabolic disease, biomarker discovery, Functional Lipidome framework	Ademowo et al. (2024); Eichelmann et al. (2024); Li et al. (2025); Han and Gross (2022)

Long‐chain PUFAs can markedly influence membrane biophysical properties. DHA, which contains six double bonds, introduces substantial conformational flexibility into phospholipid acyl chains and affects lipid packing, membrane‐domain organization, and interactions with membrane‐associated proteins. These properties are particularly important in metabolically active tissues and the nervous system, where highly unsaturated phospholipids contribute to membrane dynamics and signaling (Harayama and Shimizu 2020). In contrast, excessive accumulation of selected saturated lipid species under lipotoxic conditions may contribute to endoplasmic‐reticulum stress, mitochondrial dysfunction, ceramide accumulation, and impaired insulin signaling. These effects are dependent on lipid species, metabolic context, and cellular compartment rather than membrane saturation alone (Yang et al. 2024).

Membrane lipids also serve as reservoirs for signaling precursors. Phospholipase‐mediated release of AA, EPA, and DHA enables the enzymatic generation of diverse oxylipins and other lipid mediators involved in vascular regulation, immunity, inflammation, and inflammation resolution. AA‐derived products include both inflammatory and homeostatic mediators, whereas EPA‐ and DHA‐derived pathways generate distinct mediators with anti‐inflammatory and proresolving functions, including resolvins, protectins, and maresins (Kumar et al. 2019; Oppedisano et al. 2020; Banaszak et al. 2024). These pathways are represented schematically in Figure 2. The biological consequences of PUFA metabolism therefore depend on the precursor fatty acid, enzymatic pathway, cellular context, and mediator produced rather than on a simplistic binary distinction between n‐6 and n‐3 fatty acids.

Fatty acids also regulate intracellular signaling and transcription. Long‐chain fatty acids and their metabolites interact with PPARs and other nutrient‐sensitive transcription factors that coordinate fatty‐acid oxidation, lipid storage, mitochondrial metabolism, and inflammatory responses (Haro et al. 2019). Omega‐3 fatty acids can additionally influence FFAR4/GPR120‐dependent signaling, with experimental evidence indicating downstream modulation of inflammatory pathways and insulin sensitivity (Yang et al. 2024). These signaling mechanisms, summarized in Figure 2, provide a mechanistic link between extracellular nutrient availability, membrane lipid remodeling, and transcriptional adaptation.

Epigenetic regulation represents a further level of interaction between lipid metabolism and cellular phenotype. Fatty‐acid exposure has been associated with changes in DNA methylation and other epigenetic processes involved in metabolic and inflammatory regulation (González‐Becerra et al. 2019; Ediriweera and Gayashani Sandamalika 2025). Human supplementation studies have reported alterations in leukocyte DNA methylation following omega‐3 fatty‐acid exposure, demonstrating that the epigenome is responsive to nutritional lipid signals (Tremblay et al. 2017). However, the persistence, tissue specificity, functional significance, and causal contribution of these epigenetic changes remain incompletely established.

Short‐chain fatty acids (SCFAs) generated through microbial fermentation provide an additional example of lipid‐related epigenetic regulation. These metabolites can influence immune and metabolic pathways partly through effects on chromatin‐regulating enzymes, thereby linking intestinal microbial metabolism with host inflammatory and transcriptional responses (Kopczyńska and Kowalczyk 2024). Such mechanisms illustrate why FA‐related phenotypes should increasingly be interpreted within broader biological networks encompassing dietary exposure, host metabolism, microbial metabolites, gene regulation, and tissue‐specific lipid remodeling.

Collectively, FA signatures represent integrated molecular phenotypes connecting membrane biophysics, endogenous lipid metabolism, intracellular signaling, inflammatory regulation, and gene‐expression control. This mechanistic framework helps explain why alterations in fatty‐acid composition are associated with cardiometabolic disease, neurological dysfunction, and biological aging and provides the biological rationale for investigating FA signatures as nutritional and metabolic biomarkers. Molecular lipidomics adds structural resolution to these relationships, whereas its methodological implementation is considered separately in Section 5 to maintain conceptual clarity and avoid repetition of the analytical comparison already highlighted by the reviewer.

## 4 FA Signatures as Indicators of Health, Disease, and Biological Aging

FA signatures have evolved from relatively simple indicators of dietary fat exposure into integrated biomarkers of metabolic status, disease susceptibility, and biological aging. Unlike conventional clinical lipid measures such as total cholesterol and triglycerides, FA signatures reflect the combined influence of dietary intake, endogenous lipogenesis, fatty‐acid elongation and desaturation, β‐oxidation, lipid turnover, and tissue‐specific remodeling. The relative distribution of SFAs, MUFAs, and PUFAs, together with selected ratios and metabolic indices, can therefore provide information on both nutritional exposure and endogenous lipid regulation (Chen and Liu 2020; Ferreri et al. 2016; Almeida et al. 2021).

The biological significance of FA signatures is particularly evident in cellular membranes, where fatty‐acyl composition contributes to membrane fluidity, curvature, permeability, receptor organization, lipid‐mediated signaling, and mitochondrial function. Membrane lipid homeostasis is actively maintained despite changes in dietary lipid availability, indicating that FA patterns are not passive reflections of food intake but regulated components of cellular physiology (van Meer et al. 2008; Levental et al. 2020). Perturbation of this homeostasis can influence inflammatory signaling, insulin responsiveness, oxidative balance, cellular stress responses, and metabolic adaptation, thereby linking altered FA composition with cardiometabolic dysfunction, neurological decline, and aging‐related loss of physiological resilience (Harayama and Shimizu 2020; Ademowo et al. 2024).

Molecular lipid studies have further shown that the biological interpretation of an FA signature depends partly on the lipid species and metabolic compartments in which individual fatty acids are incorporated. Phospholipids, sphingolipids, ceramides, plasmalogens, and triacylglycerols participate in distinct structural and signaling processes and therefore provide additional context for interpreting FA‐related phenotypes (Gross 2017; Han and Gross 2022). This principle is considered here only in relation to disease and aging biology; the analytical characteristics of the respective measurement platforms are discussed separately in the dedicated analytical section. The major clinically relevant FA‐derived indices are summarized in Table 3, whereas representative disease‐associated lipid patterns are presented in Table 4.

**TABLE 3:** Clinically relevant FA signature‐derived indices and their biological interpretation. Conventional FA signature‐derived indices provide complementary information regarding long‐term dietary lipid exposure, membrane fatty acid composition, inflammatory balance, and nutritional quality. Although these indices remain valuable for nutritional assessment and cardiovascular risk evaluation, they do not capture the molecular lipid species identified by high‐resolution lipidomics. Integration of these classical indices with molecular lipid profiling may improve mechanistic interpretation and support precision nutrition and functional food research.

FA signature‐derived index	Primary analytical matrix	Biological interpretation	Major clinical and nutritional applications	References
Omega‐3 Index	Erythrocyte (red blood cell) membrane phospholipids	Reflects long‐term incorporation of eicosapentaenoic acid (EPA) and docosahexaenoic acid (DHA) into cell membranes; indicator of membrane omega‐3 status and inflammation‐resolution capacity	Cardiovascular risk assessment, healthy aging studies, monitoring dietary omega‐3 interventions	Ali et al. (2023); Cao, Yang, Guo, et al. (2024); Eichelmann et al. (2024)
Arachidonic acid (AA)/EPA ratio	Plasma phospholipids; erythrocyte membrane phospholipids	Reflects the balance between arachidonic acid‐derived pro‐inflammatory pathways and EPA‐derived inflammation‐resolving pathways	Chronic inflammation, metabolic syndrome, cardiometabolic disease, precision nutrition	Banaszak et al. (2024); Oppedisano et al. (2020); Cao, Yang, McClements, et al. (2024)
Atherogenic Index (AI)	Food lipid extracts; dietary lipid composition	Estimates the relative atherogenic potential of dietary fatty acid composition based on the balance between hypercholesterolemic and protective fatty acids	Evaluation of dietary fat quality, cardiovascular nutrition, functional food assessment	Ulbricht and Southgate (1991); Dal Bosco et al. (2024); Menotti et al. (2024)
Thrombogenic Index (TI)	Food lipid extracts; dietary lipid composition	Estimates the thrombogenic potential of dietary lipid profiles by evaluating fatty acid composition associated with platelet aggregation and thrombosis	Cardiovascular nutrition, dietary fat evaluation, functional food development	Ulbricht and Southgate (1991); Dal Bosco et al. (2024); Menotti et al. (2024)
Healthy Fatty Index (HFI)	Food matrices; dietary lipid profiles	Integrative assessment of lipid quality considering fatty acid unsaturation, PUFA/SFA balance, and overall nutritional value	Functional food development, comparative food quality assessment, nutritional evaluation	Dal Bosco et al. (2024); Cao, Yang, McClements, et al. (2024); Chen and Liu (2020)

**TABLE 4:** Disease‐associated FA signatures, lipidomic characteristics, and biological implications. Characteristic FA signatures and molecular lipidomic alterations are associated with major chronic diseases and healthy aging. Conventional FA signatures provide information on long‐term dietary lipid exposure and endogenous metabolism, whereas lipidomics reveals disease‐associated remodeling of membrane phospholipids, sphingolipids, ceramides, plasmalogens, and glycerolipids. Integrating these complementary approaches improves mechanistic understanding of membrane homeostasis, metabolic dysfunction, and biomarker discovery for precision nutrition.

Physiological condition	Characteristic FA signatures	Representative lipidomic alterations	Major biological implications	References
Cardiovascular disease (CVD)	Reduced membrane omega‐3 fatty acids (EPA and DHA); elevated omega‐6/omega‐3 ratio; increased saturated fatty acid (SFA)/polyunsaturated fatty acid (PUFA) ratio	Remodeling of phosphatidylcholine and phosphatidylethanolamine species; altered triacylglycerol (TAG) networks; pro‐inflammatory lipid mediator profiles	Endothelial dysfunction, impaired membrane homeostasis, chronic vascular inflammation, increased cardiometabolic risk	Cao, Yang, Guo, et al. (2024); Eichelmann et al. (2024); Menotti et al. (2024); Banaszak et al. (2024)
Type 2 diabetes mellitus (T2D)	Elevated SFA abundance; reduced long‐chain omega‐3 PUFAs; increased indices of endogenous lipogenesis and desaturation	Increased ceramide and diacylglycerol species; phospholipid remodeling; disruption of membrane lipid homeostasis	Insulin resistance, impaired insulin signaling, mitochondrial dysfunction, chronic low‐grade inflammation	Gaeini et al. (2025); Yang et al. (2024); Schulze et al. (2020); Ademowo et al. (2024)
Metabolic dysfunction–associated steatotic liver disease (MASLD)	Increased palmitic acid and monounsaturated fatty acids; reduced omega‐3 PUFAs; enhanced de novo lipogenesis	Hepatic triacylglycerol accumulation; altered phosphatidylcholine/phosphatidylethanolamine balance; increased lipotoxic lipid species	Hepatic lipid accumulation, endoplasmic reticulum stress, impaired β‐oxidation, progression of metabolic liver disease	Dua et al. (2025); Schulze et al. (2020); Eichelmann et al. (2024)
Cognitive decline and neurodegenerative disorders	Reduced docosahexaenoic acid (DHA); elevated omega‐6/omega‐3 ratio	Reduced plasmalogen abundance; phospholipid remodeling; altered neuroprotective lipid mediator synthesis	Neuroinflammation, impaired synaptic function, reduced membrane integrity, compromised brain resilience	Andriambelo et al. (2023); Macaron et al. (2021); Wen et al. (2024); Almeida et al. (2021)
Healthy aging and longevity	Higher long‐chain omega‐3 PUFA abundance; lower omega‐6/omega‐3 ratio; greater membrane unsaturation	Enrichment of highly unsaturated phospholipid and triacylglycerol species; reduced ceramide burden; preservation of membrane lipid diversity	Enhanced metabolic flexibility, efficient inflammation resolution, improved mitochondrial function, increased physiological resilience	Aiello et al. (2024); Ali et al. (2023); Li et al. (2025); Ademowo et al. (2024)

### 4.1 FA Signatures in Cardiometabolic Health and Metabolic Dysfunction

Cardiometabolic disorders provide some of the strongest evidence for the biological relevance of FA signatures. Altered proportions of long‐chain n‐3 PUFAs, increased abundance of selected saturated lipid species, disturbed PUFA metabolism, and changes in endogenous desaturation pathways have been associated with obesity, insulin resistance, type 2 diabetes mellitus, atherosclerosis, and cardiovascular disease. These patterns reflect interactions among dietary lipid quality, endogenous metabolism, adiposity, inflammation, and tissue remodeling rather than dietary exposure alone (Schulze et al. 2020; Cao, Yang, Guo, et al. 2024; Cao, Yang, McClements, et al. 2024).

Long‐chain n‐3 PUFAs, particularly eicosapentaenoic acid (EPA) and docosahexaenoic acid (DHA), influence cardiometabolic physiology through multiple interconnected mechanisms. Incorporation of EPA and DHA into membrane phospholipids can alter membrane organization, receptor‐associated signaling, and the substrate pool available for lipid‐mediator synthesis. Their downstream metabolites participate in inflammation‐resolution pathways and may contribute to vascular homeostasis, whereas AA gives rise to a diverse group of eicosanoids with context‐dependent inflammatory, vascular, and thrombotic functions (Oppedisano et al. 2020; Banaszak et al. 2024; Djuricic and Calder 2025). Consequently, the physiological significance of PUFA metabolism cannot be determined from the concentration of a single fatty acid alone.

Altered FA composition also intersects with insulin signaling and mitochondrial metabolism. Accumulation of specific saturated and sphingolipid species under conditions of chronic metabolic overload can contribute to lipotoxicity, endoplasmic‐reticulum stress, ceramide accumulation, mitochondrial dysfunction, and impairment of insulin‐responsive signaling pathways. Conversely, experimental evidence indicates that EPA and DHA can modulate adipose‐tissue inflammation and signaling pathways involving GPR120/FFAR4 and PPARγ, thereby providing a mechanistic link between PUFA status and insulin sensitivity (Yang et al. 2024). These observations indicate that cardiometabolic dysfunction is associated with coordinated lipid remodeling rather than with an isolated abnormality in a single FA biomarker.

Population‐level lipid studies reinforce this interpretation. Lipidomic risk scores incorporating multiple molecular species have predicted incident diabetes and cardiovascular disease independently of polygenic risk scores, indicating that lipid phenotypes contain information relevant to metabolic risk stratification (Lauber et al. 2022). Dietary intervention studies have likewise demonstrated that improving dietary fat quality produces coordinated changes in circulating lipid species associated with a more favorable cardiometabolic profile (Eichelmann et al. 2024). These findings support the biological relevance of lipid‐pattern analysis while avoiding the assumption that any individual lipid marker provides sufficient information for clinical prediction.

Established FA‐derived nutritional indices nevertheless remain useful because they provide interpretable measures of dietary lipid quality. The atherogenicity and thrombogenicity indices were developed to describe features of dietary FA composition potentially relevant to coronary risk and remain widely applied in nutritional and food‐quality research (Ulbricht and Southgate 1991). Long‐term evidence from the Seven Countries Study has further linked these dietary indices with cardiovascular mortality (Menotti et al. 2024). More recently, the Healthy Fatty Index has been introduced to provide additional characterization of the lipid quality of animal‐derived foods (Dal Bosco et al. 2024). Such indices should, however, be interpreted within the broader clinical and metabolic context rather than as independent diagnostic tools.

### 4.2 FA Signatures in Neuroinflammation, Cognitive Function, and Brain Aging

The central nervous system has distinctive lipid requirements because neuronal membranes contain substantial quantities of highly unsaturated fatty acids, particularly DHA. Appropriate membrane lipid composition contributes to synaptic vesicle dynamics, receptor function, ion‐channel activity, membrane trafficking, neuronal signaling, and structural plasticity. Disturbances in PUFA metabolism and membrane remodeling have therefore been investigated in relation to neuroinflammation, cognitive impairment, and neurodegenerative disorders (Harayama and Shimizu 2020; Macaron et al. 2021).

DHA is particularly relevant to brain aging because it contributes to neuronal membrane properties and serves as a precursor for lipid mediators involved in neuroprotective and inflammation‐resolving pathways. Reduced DHA availability or altered long‐chain n‐3 PUFA metabolism may affect neuronal membrane organization, mitochondrial function, and inflammatory regulation. Evidence further suggests that n‐3 PUFAs may influence blood–brain barrier integrity and biological processes associated with glymphatic function, providing additional mechanisms through which lipid status may contribute to maintenance of brain homeostasis during aging (Wen et al. 2024).

Associations between n‐3 PUFA status and brain morphology have been described in cognitively healthy older adults, although available studies remain heterogeneous in design, exposure assessment, population characteristics, and neuroimaging outcomes (Macaron et al. 2021). Randomized trials of omega‐3 supplementation have likewise yielded variable cognitive outcomes. Differences in baseline n‐3 status, age, disease stage, dose, formulation, intervention duration, genetic background, habitual diet, and metabolic phenotype may contribute to this heterogeneity (Andriambelo et al. 2023; Dyall and Plourde 2025).

These observations have important implications for nutritional intervention studies. Participants with low baseline long‐chain n‐3 PUFA status may respond differently from those whose baseline status is already adequate, while established neurodegenerative pathology may further modify responsiveness. Future trials should therefore combine objective assessment of FA status with clinically relevant cognitive, functional, imaging, and molecular outcomes. Such stratification may help identify populations in whom targeted nutritional modification is most likely to provide measurable benefit.

### 4.3 FA Signatures and Lipidomics in Biological Aging and Precision Nutrition

Aging is accompanied by coordinated changes in lipid metabolism, membrane composition, mitochondrial function, inflammatory regulation, redox homeostasis, and metabolic flexibility. FA signatures can integrate dietary exposure with endogenous fatty acid metabolism and tissue‐specific remodeling and may therefore contribute to characterization of biological aging phenotypes. Studies across adult populations and long‐lived groups have identified associations between FA composition, PUFA status, inflammatory markers, and healthy aging, although these relationships remain influenced by diet, metabolic status, genotype, and other lifestyle factors (Ali et al. 2023; Aiello et al. 2024).

Membrane and mitochondrial lipid remodeling provides an important mechanistic link between FA metabolism and aging. Mitochondrial membranes require tightly regulated lipid composition to maintain electron transport, oxidative phosphorylation, and membrane potential, whereas age‐related disturbances in lipid homeostasis may contribute to oxidative stress and impaired metabolic function (Levental et al. 2020; Xie et al. 2021). Omega‐3 fatty acids may also influence pathways involving sirtuins and PGC‐1α that regulate mitochondrial biogenesis and energy metabolism (Son et al. 2021). Conversely, persistent nutrient excess and accumulation of lipotoxic lipid species can promote insulin resistance and metabolic inflexibility.

FA composition also influences inflammatory regulation, but the biological relationship between n‐3 and n‐6 PUFAs should not be reduced to a simple pro‐inflammatory versus anti‐inflammatory dichotomy. AA, EPA, and DHA serve as substrates for multiple lipid‐mediator pathways, and their effects depend on tissue availability, enzymatic metabolism, and the biological context in which mediators are generated. EPA and DHA contribute to specialized proresolving mediator biosynthesis, whereas AA generates diverse eicosanoids with inflammatory, vascular, and homeostatic functions (Schulze et al. 2020; Sarajlic et al. 2023; Djuricic and Calder 2025). This complexity is particularly relevant when interpreting FA biomarkers in aging populations.

Lipidomics further indicates that biological aging is associated with coordinated changes in molecular lipid architecture rather than isolated alterations in individual fatty acids. Variation in triacylglycerol chain length and saturation, together with changes in phospholipids and plasmalogens, has been associated with metabolic and healthy‐aging phenotypes (Li et al. 2025; Ferreri et al. 2023). These observations suggest that molecular lipid composition may complement conventional FA measurements when characterizing biological aging.

FA metabolism may additionally interact with epigenetic regulation. Fatty acids and their metabolites can influence nutrient‐sensitive transcriptional pathways and have been associated with changes in DNA methylation and other chromatin‐related processes (Haro et al. 2019; González‐Becerra et al. 2019; Ediriweera and Gayashani Sandamalika 2025). Omega‐3 supplementation has also been reported to alter leukocyte DNA methylation (Tremblay et al. 2017). However, the tissue specificity, persistence, and functional consequences of these changes remain insufficiently established. Similarly, associations between PUFA status and telomere length provide an additional potential link between lipid metabolism and aging but remain observational and should not be interpreted as evidence of causality (Dhillon et al. 2021).

For precision nutrition, these findings support the use of FA signatures and lipidomics as complementary components of biological phenotyping rather than as standalone measures of biological age. Prospective studies integrating lipid composition with metabolic, inflammatory, functional, and dietary measures are required to determine whether specific lipid phenotypes predict aging trajectories or primarily reflect established physiological changes.

### 4.4 Nutrition as Routine Primary Prevention

The relationship between lipid metabolism and aging has important implications for primary prevention. Improving overall dietary quality, ensuring adequate essential fatty‐acid intake, and avoiding chronic energy excess can influence lipid metabolism throughout the life course. Controlled dietary studies demonstrate that changes in dietary fat quality can produce coordinated remodeling of circulating lipid profiles associated with cardiometabolic risk (Eichelmann et al. 2024). These findings support sustained dietary improvement rather than reliance on isolated nutrients or supplementation after metabolic dysfunction has developed.

The response to EPA and DHA supplementation is heterogeneous and depends on baseline status, dose, formulation, intervention duration, background diet, and individual metabolic characteristics (Andriambelo et al. 2023; Djuricic and Calder 2025). Accordingly, precision nutrition should consider baseline FA status when evaluating nutritional insufficiency or potential responsiveness. Biochemical FA measurements can complement dietary assessment but cannot independently reconstruct dietary intake because endogenous synthesis, absorption, metabolism, genotype, disease, medication use, and tissue turnover also influence FA composition.

Foodomics provides an additional translational dimension by linking molecular food composition with biological responses. Mass‐spectrometry‐based approaches can characterize food lipids, processing‐related modifications, and molecular diversity that may influence nutritional quality and physiological effects (Herrero et al. 2012; Mahato et al. 2024; Martakos et al. 2024). Integrating food lipidomics with human lipidomics could therefore help clarify why foods with broadly similar total‐fat or PUFA contents may produce different metabolic responses.

A major priority is prospective validation. Longitudinal studies should determine whether specific FA and lipidomic patterns precede functional decline, predict metabolic resilience, or primarily reflect consequences of aging. Such studies should account for major determinants of lipid metabolism, including sex, adiposity, habitual diet, physical activity, medication use, metabolic disease, and genetic variation (Chaaba et al. 2023; Wang et al. 2023).

The objective should therefore not be to define a single universal lipid biomarker of aging, but to identify reproducible and clinically meaningful lipid phenotypes associated with favorable or adverse aging trajectories and to establish whether these phenotypes are modifiable through nutritional intervention. Within this evidence‐based framework, FA signatures provide a useful bridge between dietary exposure, membrane biology, lipid metabolism, and physiological adaptation, while lipidomics offers additional molecular resolution for biomarker discovery and precision nutrition.

## 5 Analytical Frontiers and Integrative Omics: Expanding the Functional Lipidome

**FIGURE 3:**
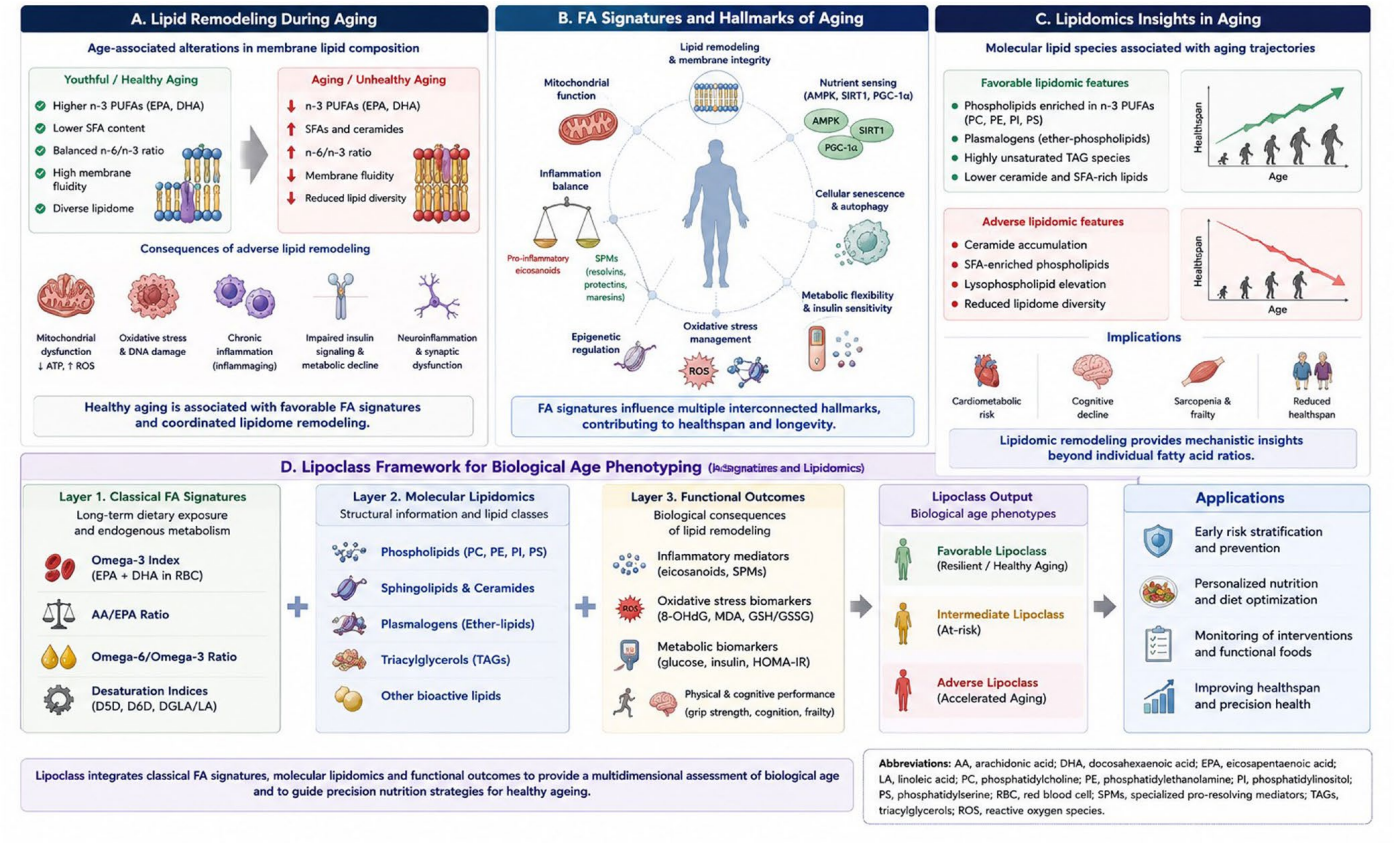
Systems‐level integration of FA signatures, molecular lipidomics, and complementary omics in nutritional lipid research. FA‐related phenotypes can be interpreted within a multidimensional network incorporating molecular lipid species, genomics, transcriptomics, proteomics, metabolomics, foodomics, microbiome‐related information, and computational analysis. Integration of these complementary layers enables systems‐level investigation of membrane remodeling, inflammatory regulation, mitochondrial metabolism, cardiometabolic health, biological aging, and interindividual responses to dietary exposure. Computational approaches, including machine learning and artificial intelligence, may facilitate pattern recognition and multidimensional data integration but require rigorous validation and biological interpretability. This integrative functional‐lipidome perspective may support biomarker discovery, precision nutrition, functional‐food research, and preventive health strategies, although prospective validation is required before clinical implementation.

Advances in analytical chemistry and lipidomics have expanded fatty‐acid research from measurement of individual fatty acids to characterization of their molecular lipid context. Conventional gas chromatography–fatty acid methyl ester (GC–FAME) analysis remains essential for quantitative fatty‐acid profiling and nutritional indices, whereas liquid chromatography–mass spectrometry (LC–MS) provides complementary information on intact phospholipids, sphingolipids, ceramides, plasmalogens, glycerolipids, and other molecular species (Gross 2017; Han and Gross 2022; Fedorova et al. 2026). Together, these approaches enable FA signatures to be interpreted in relation to membrane organization, metabolic pathways, and physiological responses. The principal analytical and biological relationships are summarized in Figure 3.

### 5.1 From FA Profiling to Molecular Lipidomics

GC–FAME remains particularly valuable for determining individual fatty‐acid composition, membrane FA profiles, desaturation indices, and established nutritional indices. LC–MS, by contrast, places fatty acids within their intact molecular lipid context and can reveal remodeling of phospholipids, sphingolipids, ceramides, plasmalogens, and triacylglycerols that may not be apparent from aggregate FA measurements (Han and Gross 2022; Züllig and Köfeler 2021; Ademowo et al. 2024; Eichelmann et al. 2024). Thus, these platforms should be considered complementary rather than hierarchical.

Structural resolution remains an important limitation. Untargeted LC–MS workflows may incompletely resolve positional isomers, double‐bond locations, and geometric configurations, despite their potentially distinct biological properties. Hexadecenoic‐acid positional isomers illustrate this challenge and demonstrate why conventional FA analysis remains important alongside molecular lipidomics (Sansone et al. 2013; Scanferlato et al. 2019). Plasmalogen isomerism and oxidative modifications provide further examples of the need for rigorous structural characterization (Ferreri et al. 2023).

Analytical interpretation is also dependent on preanalytical and biological factors. Plasma, whole blood, erythrocytes, adipose tissue, and isolated membranes represent distinct lipid compartments and should not be treated as interchangeable matrices (Ferreri et al. 2016). Sample handling, extraction, derivatization, internal standards, instrument calibration, lipid annotation, and computational processing can further influence results. Standardized procedures, appropriate reference materials, quality assurance, and laboratory quality‐management systems are therefore essential for reproducible research and eventual clinical translation (Takahashi 2026).

### 5.2 Multi‐Omics Integration of Lipid Metabolism

FA signatures acquire greater biological meaning when integrated with complementary molecular layers. Genomic variation in fatty‐acid desaturases and elongases contributes to interindividual differences in PUFA metabolism and may influence responses to dietary intervention (Chaaba et al. 2023; Wang et al. 2023). At the transcriptional level, nutrient‐sensitive regulators, including peroxisome proliferator‐activated receptors and sterol regulatory element‐binding proteins, coordinate lipid synthesis, oxidation, inflammation, and metabolic adaptation (Haro et al. 2019).

Metabolomics and proteomics provide additional information on the consequences of lipid remodeling, including mitochondrial substrate utilization, β‐oxidation, oxylipin formation, oxidative stress, and inflammatory signaling. These complementary measurements help distinguish changes in FA abundance from alterations in downstream metabolic activity (Kaliannan et al. 2019; Sarajlic et al. 2023). Epigenetic analyses provide another layer through which dietary fatty acids and their metabolites may influence transcriptional regulation, although the persistence and functional significance of these effects remain incompletely established (González‐Becerra et al. 2019; Tremblay et al. 2017; Ediriweera and Gayashani Sandamalika 2025).

Accordingly, the principal value of multi‐omics integration lies not in accumulating molecular measurements but in connecting dietary exposure, genetic variation, enzyme activity, lipid remodeling, signaling intermediates, and physiological outcomes within biologically interpretable pathways. Recent developments in lipidomics emphasize the need for standardized workflows, interoperable resources, rigorous annotation, and reproducible analytical frameworks to support this transition (Fedorova et al. 2026).

### 5.3 Foodomics and Dietary Lipid Exposure

Understanding host lipid phenotypes also requires characterization of their dietary sources. Foodomics integrates advanced analytical approaches to characterize food composition, processing‐related changes, authenticity, and bioactive constituents (Herrero et al. 2012). Food lipidomics can identify molecular lipid species that are not adequately represented by total‐fat or conventional FA measurements and can therefore provide greater resolution of dietary lipid exposure.

Molecular lipid composition may vary substantially among foods according to species, production conditions, processing, extraction, and storage (Martakos et al. 2024). Characterization of alternative and marine‐derived lipid resources further demonstrates how molecular profiling can guide the recovery and development of nutritionally valuable lipid ingredients (Martakos et al. 2026). Integrating food lipidomics with human lipid profiling may therefore clarify how specific dietary lipid structures influence circulating and tissue lipid remodeling and could strengthen evidence‐based functional‐food development (Mahato et al. 2024).

### 5.4 Computational Lipidomics and Predictive Modeling

The high dimensionality of lipidomic datasets creates opportunities for multivariate statistics and machine‐learning approaches but also introduces risks of overfitting, cohort‐specific bias, and limited reproducibility. Lipidomic risk scores have demonstrated associations with incident diabetes and cardiovascular disease that provide information beyond polygenic risk scores, illustrating the potential value of molecular lipid patterns for disease prediction (Lauber et al. 2022). Nevertheless, predictive performance within discovery cohorts does not establish clinical utility.

Computational models integrating FA signatures with lipidomic, dietary, clinical, and genetic variables will require independent external validation, transparent reporting, biologically interpretable features, and prospective evaluation. AI should therefore be regarded as a developing analytical tool rather than an established clinical decision‐making technology. Standardized datasets and reproducible computational workflows will be essential for determining whether such approaches provide meaningful improvements over established nutritional and clinical measures (Fedorova et al. 2026).

### 5.5 Translational Perspective

The evidence supports a complementary analytical framework in which conventional FA profiling provides quantitative information on fatty‐acid composition, whereas molecular lipidomics, foodomics, and multi‐omics approaches provide progressively greater biological context (Figure 3). The functional lipidome is used here as a descriptive framework for integrating these layers, rather than as a validated clinical model. Its utility will depend on demonstrating that molecular integration improves biological interpretation, biomarker performance, or prediction of nutritional responses beyond established measurements.

Future progress should therefore prioritize structural lipid identification, harmonized analytical procedures, appropriate biological matrices, reproducible computational pipelines, and validation in independent populations. The goal is not simply to increase molecular coverage but to establish which lipid features are reproducible, biologically informative, and clinically actionable. This evidence‐based progression could strengthen understanding of diet–lipid interactions and support more precise nutritional and functional‐food research while avoiding premature translation of exploratory lipidomic patterns into clinical practice.

## 6 Multi‐Omics Integration, Microbiome Interactions, and Epigenetic Regulation

The biological interpretation of FA signatures increasingly requires integration across molecular layers rather than expansion of individual measurements. Dietary exposure interacts with host genetics, endogenous lipid metabolism, cellular regulation, microbial activity, and environmental factors to produce heterogeneous lipid phenotypes. Multi‐omics approaches can help resolve these relationships by connecting lipid composition with upstream regulatory mechanisms and downstream metabolic consequences (Kaliannan et al. 2019; Fedorova et al. 2026).

### 6.1 Genetic and Metabolic Determinants of FA Signatures

Host genetic variation contributes substantially to interindividual differences in FA metabolism. Variants affecting fatty‐acid desaturation and elongation can modify the endogenous conversion of precursor fatty acids into long‐chain PUFAs and consequently influence circulating and membrane FA composition. Variation within the FADS and ELOVL pathways may therefore contribute to differences in both baseline lipid profiles and responses to dietary intervention (Chaaba et al. 2023; Wang et al. 2023).

These genetic effects interact with transcriptional and metabolic regulation. Fatty acids influence nutrient‐sensitive transcriptional pathways, whereas changes in gene expression reciprocally regulate lipid synthesis, oxidation, transport, and remodeling (Haro et al. 2019). Integrating lipidomics with transcriptomics and metabolomics may therefore help distinguish primary metabolic responses from secondary adaptations and clarify whether particular lipid phenotypes precede or accompany metabolic dysfunction. Such approaches are more informative than interpreting FA signatures independently of their underlying regulatory context.

### 6.2 Microbiome–Lipid Interactions

The gut microbiome represents an additional determinant of host lipid metabolism. Dietary lipid composition can alter microbial ecology and metabolic activity, while microbial metabolites may influence intestinal barrier function, inflammatory signaling, hepatic metabolism, insulin sensitivity, and host lipid handling. Evidence from metabolic‐disease research supports interactions among microbial metabolism, insulin resistance, and host lipid regulation, although their magnitude and direction depend on dietary context, host phenotype, medication exposure, and baseline microbial composition (Dua et al. 2025).

Microbially derived metabolites may provide mechanistic links between intestinal ecology and systemic metabolism. SCFAs, for example, participate in immune‐metabolic signaling and epigenetic regulation relevant to metabolic health and chronic inflammation (Kopczyńska and Kowalczyk 2024). Other microbial products may influence bile‐acid metabolism, intestinal lipid absorption, hepatic substrate utilization, and inflammatory pathways. However, evidence remains insufficient to establish how consistently these processes modify circulating or membrane FA signatures. Controlled dietary studies integrating microbiome profiling with lipidomics and metabolomics are therefore needed to distinguish causal microbial effects from associations driven by shared dietary or host factors.

Interindividual variability is particularly important. Similar dietary interventions can produce different microbial and metabolic responses according to baseline microbiota, genotype, habitual diet, age, metabolic status, and medication use. Repeated within‐person measurements and controlled interventions will consequently be more informative than cross‐sectional comparisons for defining microbiome‐associated lipid phenotypes.

### 6.3 Epigenetic Regulation and Nutritional Memory

Epigenetic mechanisms provide another potential link between dietary lipid exposure and longer‐term metabolic phenotypes. Fatty acids and their metabolites can interact with pathways involved in DNA methylation, chromatin accessibility, and transcriptional regulation, with potential consequences for inflammatory and metabolic homeostasis (González‐Becerra et al. 2019; Ediriweera and Gayashani Sandamalika 2025). Human intervention studies have reported alterations in leukocyte DNA methylation following omega‐3 supplementation, supporting the biological plausibility of diet‐responsive epigenetic regulation (Tremblay et al. 2017).

However, the interpretation of these findings requires caution. The persistence, tissue specificity, and functional consequences of diet‐associated epigenetic changes remain incompletely established. Peripheral‐blood epigenetic profiles may not reflect regulatory events in metabolically relevant tissues, and observed methylation differences may represent consequences rather than mediators of altered lipid metabolism. Accordingly, epigenetic associations should not be interpreted as evidence that modification of a particular FA directly produces durable biological‐aging effects.

### 6.4 Establishing Causality Through Integrated Longitudinal Studies

A central challenge across these biological layers is establishing temporal and causal relationships. FA signatures may influence transcriptional, inflammatory, or metabolic pathways while simultaneously being altered by those processes. Similarly, microbiome composition and epigenetic regulation can both respond to and influence dietary and metabolic states. Prospective intervention studies with repeated molecular measurements are therefore needed to distinguish exposure, response, and consequence.

Where feasible, stable‐isotope tracing can provide additional information on fatty‐acid synthesis, incorporation, turnover, and tissue‐specific flux. Combining these measurements with lipidomics, genomics, transcriptomics, metabolomics, microbiome profiling, and epigenomics may identify biological pathways that explain interindividual differences in nutritional responses more effectively than any single omics platform.

The objective should not be indiscriminate accumulation of high‐dimensional datasets but identification of reproducible, biologically coherent pathways linking dietary exposure to lipid remodeling and physiological outcomes. Interpretation should preserve information on biological compartment, temporal sequence, and tissue specificity because circulating lipid profiles cannot necessarily be assumed to represent lipid metabolism in the liver, adipose tissue, skeletal muscle, brain, or other target organs.

### 6.5 Translational Priorities

Future investigations should prioritize longitudinal cohorts and controlled nutritional interventions in which dietary exposure, FA composition, molecular lipid species, microbiome characteristics, epigenetic regulation, and clinically relevant outcomes are assessed within the same individuals. Such designs could clarify whether particular lipid phenotypes predict metabolic resilience or differential nutritional responsiveness and whether they are modifiable through dietary intervention.

International lipidomics initiatives further emphasize harmonized analytical workflows, interoperable datasets, reproducible annotation, and cross‐disciplinary integration as prerequisites for translational application (Fedorova et al. 2026). Thus, multi‐omics integration should be viewed as a means of strengthening mechanistic inference rather than as an endpoint in itself. Establishing causal relationships among diet, host genetics, microbial metabolism, epigenetic regulation, and lipid remodeling represents an important next step toward biologically grounded precision nutrition.

## 7 Conclusion

FA signatures have evolved from descriptive measures of dietary fat intake into mechanistically informative indicators of lipid metabolism, membrane organization, and physiological adaptation. By integrating dietary exposure with endogenous fatty acid synthesis, desaturation, elongation, oxidation, and tissue‐specific remodeling, FA signatures provide a biologically relevant representation of metabolic status beyond conventional lipid biomarkers. Increasing evidence indicates that membrane fatty acid composition influences cellular signaling, inflammatory regulation, mitochondrial function, and gene expression, linking dietary lipid quality with cardiometabolic health, cognitive function, and healthy aging. Advances in lipidomics further demonstrate that biological effects depend not only on fatty acid composition but also on the molecular lipid species and classes in which fatty acids are incorporated. Integrating conventional fatty acid profiling with lipidomics can therefore improve mechanistic interpretation and biomarker discovery. Important challenges remain, including analytical standardization, tissue‐specific validation, population diversity, and causal inference. Longitudinal intervention studies and rigorous multi‐omics integration will be essential to establish clinical utility. Collectively, FA signatures provide a promising framework linking nutrition, membrane biology, and metabolic health, with potential to strengthen precision nutrition and evidence‐based functional food development.

## Author Contributions

**Farzana Mim:** writing – review and editing, resources. **Md. Selim Reza:** writing – review and editing, writing – original draft. **Mohammad Nazrul Islam Bhuiyan:** conceptualization, visualization, validation, writing – review and editing, formal analysis, supervision, resources, writing – original draft.

## Funding

The authors have nothing to report.

## Ethics Statement

This study does not have an impact on animal or human health.

## Conflicts of Interest

The authors declare no conflicts of interest.

## Data Availability

Data sharing not applicable to this article as no datasets were generated or analysed during the current study.
